# Use of a gamified website to increase pain neurophysiology knowledge and improve satisfaction and motivation among students studying for a degree in physiotherapy: a quasi-experimental study

**DOI:** 10.1186/s12909-022-03457-w

**Published:** 2022-05-20

**Authors:** Fran Valenzuela-Pascual, Judith Pàmies-Fabra, Ester García-Martínez, Oriol Martínez-Navarro, Carolina Climent-Sanz, Montserrat Gea-Sánchez, Jordi Virgili-Gomà, Francesc Rubí-Carnacea, Maria Garcia-Escudero, Joan Blanco-Blanco

**Affiliations:** 1 Department of Nursing and Physiotherapy, The University of Lleida, Montserrat Roig, 2, 25198 Lleida, Spain; 2 Group for the Study of Society Health Education and Culture, GESEC, University of Lleida, Lleida, Spain; 3 Health Care Research Group, GRECS, Biomedical Research Institute of Lleida, IRBLleida, Lleida, Spain; 4 Department of Computer Science and Industrial Engineering, The University of Lleida, Lleida, Spain; 5 Faculty of Medicine and Health Sciences, Universidad Católica de Valencia San Vicente Mártir, Valencia, Spain

**Keywords:** ICT, Gamification, Motivation, Degree in physiotherapy, Teaching–learning, Neurophysiology of pain

## Abstract

**Background:**

The scientific evidence highlights the difficulties that healthcare professionals experience when managing patients with chronic pain. One of the causes of this difficulty could be related to the acquired training and the lack of knowledge about the neurophysiology of pain. In the present study, we assessed the effectiveness of a gamified web platform in acquiring knowledge about pain neurophysiology and determining the satisfaction and motivation of students of the Degree in Physiotherapy at the University of Lleida.

**Methods:**

A quasi-experimental study was carried out with a sample of 60 students who had access to a gamified web platform that included notes, videos, and clinical cases prepared by the teaching staff and was based on a previous study that included patients and healthcare professionals.

**Results:**

The results show that after the intervention, there was a statistically significant increase in knowledge about the neurophysiology of pain, and the effect size was in the desired area of ​​effect. Likewise, many students considered that their motivation had increased as a result of the methodology used in the present study.

**Conclusions:**

The results support the use of this methodology to promote knowledge about the neurophysiology of pain while improving students’ motivation.

## Background

Pain neurophysiology is essential knowledge in the study and understanding of painful processes and experiences. Among them, we can highlight chronic pain, which is defined as pain that persists or recurs for more than three months [1]. This type of pain is present in many chronic diseases, where pain is often the main complaint of patients and can even be considered a disease in itself, as in the cases of fibromyalgia syndrome (FMS) and chronic low back pain (CLBP) [2].

According to a study carried out by Pérez Sánchez et al. (2006), 19% of Europeans experience chronic pain, with back pain representing 24% of this number. In Spain, Català et al. (2002) estimated a very similar proportion (17%), noting that it was more common among women. This statement has been corroborated by other studies of international scope [3–6]. In Catalonia in 2018, 22% of the population aged 15 or higher suffered from chronic back or lumbar pain, 17.5% suffered from chronic cervical pain, and 20.5% suffered from rheumatic or locomotor system diseases [7].

Several studies show the difficulties that health professionals face in managing patients with chronic pain, as in the case of FMS [6, 8–11]. Several studies attribute these difficulties to the lack of knowledge health professionals have concerning chronic pain [12–14]. The literature shows that, in most health science degrees, the curricula present few training hours on pain, which leads to a significant lack of knowledge for future health professionals [15, 16]. This lack of knowledge affects the health care of those suffering from chronic pain. For example, patients with FMS have been shown to receive ineffective and erroneous treatments [11, 12, 17, 18], and it takes a long time for them to be correctly diagnosed [14]. All this worsens patients’ painful experiences and diminishes their quality of life, affecting biological, psychological, and social aspects [5, 19]. It is also necessary to mention the high economic cost this creates for the public health system, which fluctuates between 2.2 and 2.8% of the Spanish gross domestic product. Between 13 and 16 billion euros are spent annually due to chronic pain [20], increasing health systems costs [5, 13].

Therefore, when the Degree in Physiotherapy at the University of Lleida (UdL) was introduced, the authors were mindful of the recommendations of the International Association for the Study of Pain (IASP) [21] regarding the incorporation of a specific curriculum on pain. However, learning about the neurophysiology of pain remains a complex and challenging process for our students. This part of the syllabus is given in the course “Physiotherapy in Clinical Specialities”, where we work on the contents of physiotherapy applied to chronic processes, specifically on the neurophysiology of pain and the chronification of musculoskeletal pain. As an example of the above, a study carried out at the University of Toronto using a “Pain Education Interprofessional Resource” online significantly increased pain neurophysiology knowledge in their students [22]. However, this study did not consider student motivation.

On the other hand, the use of information and communication technologies (ICTs) in the teaching world has been steadily gaining ground. Gamification is a learning technique that uses entertainment in non-playable environments [23]. In the case of health education, Allam et al. [24] investigated the effect of gamification on a website on physical activity for patients with rheumatoid arthritis showing that gamification alone or combined with social support significantly increased physical activity. Likewise, gamification has proven effective in the education sector, both in primary and higher education [25, 26]. As some authors state, the success of this type of game lies in the innovation and motivation they offer to the people who use them [23, 25–28].

For this reason, it is believed that the use of gamification can contribute to better training of students in pain neurophysiology, which is necessary to reverse the negative consequences of the mishandling of patients with chronic pain in all societies.

## Methods

### Aim

The aim of this study was to assess the effectiveness of a gamified web platform in acquiring knowledge about pain neurophysiology and determining the levels of satisfaction and motivation of physiotherapy students regarding the platform.

### Research design

This is a quasi-experimental study with a single experimental group in which the pre- and post-intervention knowledge of students, as well as the motivation of the post-intervention students, were analysed.

This study hypothesizes that using a gamified web platform increases knowledge acquisition and improves students' motivation in UdL’s physiotherapy degree and sports sciences-physiotherapy and physiotherapy-nursing double degree programs.

### Participants

The study had a sample of 60 subjects composed of students in the “Physiotherapy in Clinical Specialities” course. These students were enrolled in physiotherapy and double degree sport sciences-physiotherapy and physiotherapy-nursing degree programs. The sample was composed of 30 men and 30 women.

### Intervention process

The intervention was carried out online. The study subjects had access to a gamified web platform accessible from any electronic device to view explanatory videos, download PDF material, or take online questionnaires (quizzes). To resolve issues, students had the option of accessing the professor in three ways: through the forum of the UdL virtual campus, by direct e-mail to the professor, or by a videoconference.

#### Gamified Web platform

To develop both the web platform and the educational material, professionals from different fields, such as physiotherapy, nursing, sociology, medicine, and computer engineering, participated. The website was developed using Drupal as the content management system. Some modules of the management system, including those related to questionnaires and video tutorials, were modified and adapted to the project's needs. This helped increase the platform’s versatility, resulting in better utilization of most systems (i.e., the registration modules by adding security for data available through an advanced encryption standard, the synchronisation modules, and mass mailings).

**Fig. 1:**
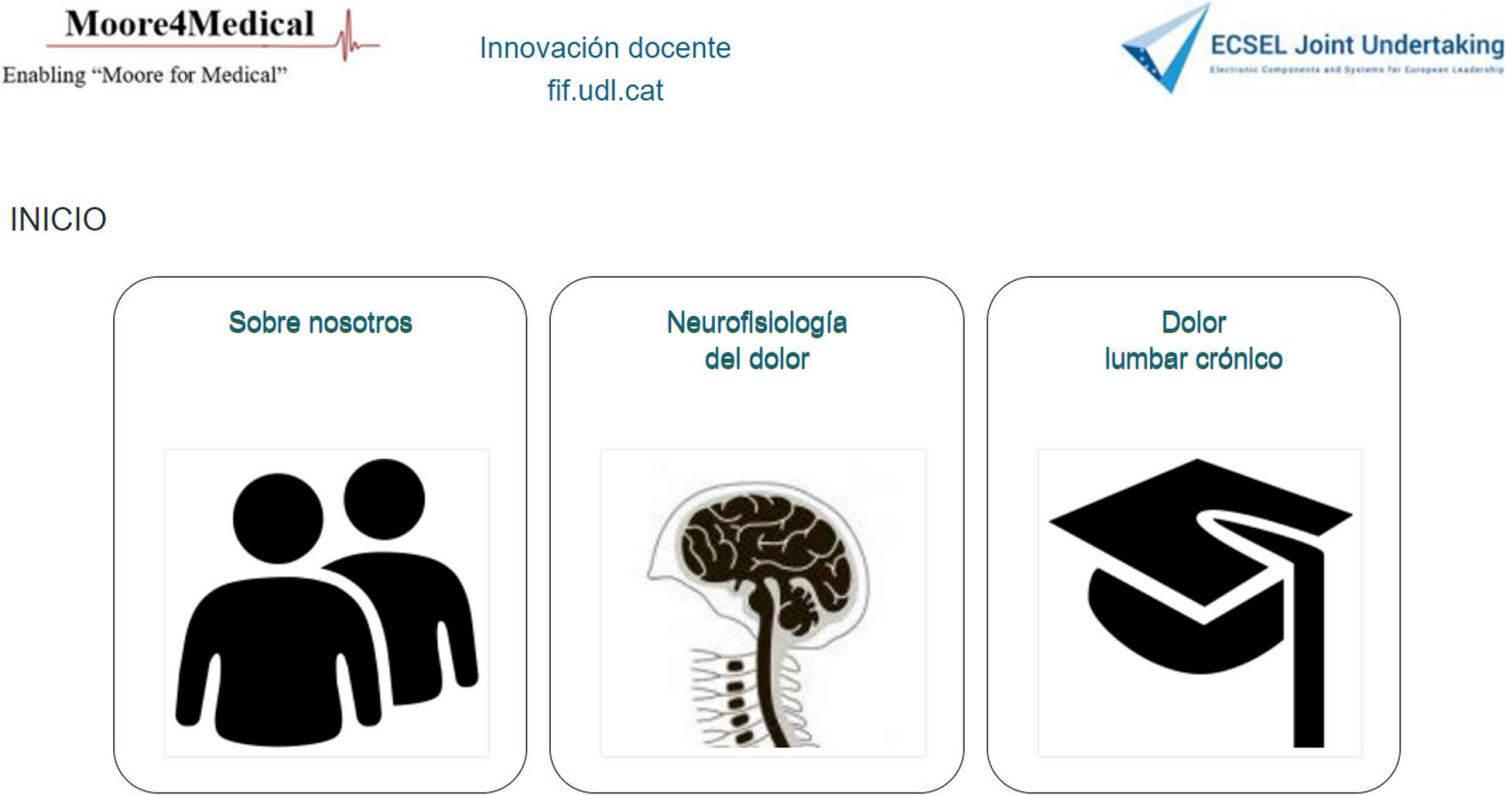
The main screen of the gamified web platform

The students were provided with individual access to a gamified web platform with all the available material to acquire knowledge of pain neurophysiology. The main screen of the gamified web platform (Fig. 1) contained an information sheet about the study. The gamified web platform contained five sections (Table 1).

**Table 1:** Sections, categories and content of the gamified web platform

Sections	Categories	Content			
*Notes*	Basic neurophysiology	Taxonomy of pain			
		Peripheral mechanisms related to the pain process			
		Central mechanisms of nociceptive transmission: dorsal horn of the spinal cord			
		Central mechanisms of nociceptive transmission: segmental and supraspinal pain modulation			
		Pain and emotional state			
	Advanced neurophysiology	The biopsychosocial model and the neurophysiology of pain			
		Taxonomy of pain			
		Neurophysiology of pain: pain modulation			
		Cognitive and emotional factors related to downward pain modulation			
		Neurobiological and psychosocial factors that mediate relationship between poor quality sleep and pain			
	Fibromyalgia	Fibroscepticism		Fibromyalgia syndrome	
	Arthropathies	Notes and a presentation on arthropathies			
	Scientific articles	Continuous passive motion following total knee arthroplasty in people with arthritis (Review) by Harvey et al. (2014)			
		Cryotherapy following total knee replacement (Review) by Adie et al. (2012)			
		Psychological evaluation of Olivares and Cruzado's pain (2008)			
		Physiotherapy interventions for shoulder pain (Review) by Green et al. (2003)			
		Reconceptualising pain according to modern pain science de Moseley (2007)			
		Complex Regional Pain Syndrome by Cuenca et al. (2012)			
		The Effect of Neuroscience Education on Pain, Disability, Anxiety, and Stress in Chronic Musculoskeletal Pain by Louw et al. (2012)			
	Flag systems	Red flags		Orange flags	
		Yellow flags		Blue/Black flags	
*Videos*	Basic neurophysiology	Introduction to neurophysiology			
		Three explanatory videos on basic neurophysiology			
	Advanced neurophysiology	A video explaining the advances in the neurophysiology of pain			
	Pain and Sleep	Two videos explaining the relationship between pain and sleep			
*Chronic low back pain*	Pain Modulation		Origin and causes of chronic low back pain		Relationship between pain and stress
	Relationship between pain and physical activity		Benefits of physical activity		
*Clinical Cases*	Jaw pain		Fibromyalgia		
	Rotator cuff		Femoral neck fracture		
	Groin pain		Chest pain		
	Anterior knee pain		Chronic low back pain I		
	Ankle sprain		Chronic low back pain II		
*Quizz*	Questions about the neurophysiology of pain I				
	Questions about the neurophysiology of pain II				
	Questions about the neurophysiology of pain III				
	Questions about the neurophysiology of pain IV				

To reinforce our students´ motivation and participation, we used gamification techniques (defined as elements forming part of the design of video games but used in a different context) [29, 30]. Two sections of the web platform were gamified, the section on “chronic low back pain” and the quiz section.

- 1) The “Notes” section was divided into eight blocks: basic neurophysiology, advanced neurophysiology, chronic pain in clinical practice, fibromyalgia, arthropathies, scientific articles (English), and assessment systems for serious pathologies related to low back pain and psychosocial aspects (flag system). Each of the blocks contained its specific material.
- 2) In the “Videos” section, there were three categories. The “basic neurophysiology” category had an introductory video and three educational videos. The “advanced neurophysiology” category included a video on the advances in the neurophysiology of pain. Finally, the “pain and sleep” category included two educational videos on the relationship between pain and sleep.
- 3) The section on “chronic low back pain” contained five educational videos: pain modulation; origin and causes of CLBP; the relationship between pain and stress; the relationship between pain and physical activity; and the beneficial effects of physical activity. Specific cuts were made to the videos so that short videos were generated to respond in a more particular way to the statements selected by the students after watching them. Once the student visualized the video "Presentation", a series of messages appeared on the screen. The student was asked to select the statement related to back pain, with which he agreed. Each message was associated with a different aspect of low back pain and was a hyperlink that led to a specific video connected to that statement. For example, if the student had clicked on the statement "Pain is always in the brain and it is the brain that decides whether to feel pain or not", a new screen would have appeared with video 2 “Pain modulation”.
  In this way, we used gamification through personalized tasks that allowed the student to use the metaphor of the trip (the narrative as a game dynamic) to feel that he was managing his path. Therefore, each student watched the videos in a particular order that depended on the statement selected at each moment. Thus, each student followed a different and personalized path thanks to the gamification of the website.
- 4) In the section on “clinical cases”, there were clinical cases with answers on jaw pain, rotator cuff pain, groin pain, knee pain, ankle sprains, fibromyalgia, femoral neck fractures, chest pain, and finally, two clinical cases on CLBP.
- 5) The “quiz” section contained four links that gave students access to four questionnaires on the neurophysiology of pain, ordered from least to most difficult. The authors developed these four questionnaires specifically for this study. A virtual platform called Quizziz (Quizizz — The world’s most engaging learning platform) was used to contribute to game enjoyment [31].

Each questionnaire consisted of ten multiple-choice questions, which could either contain single or multiple answers; in both cases, the students had 30 s to answer. In this quiz section, we have used different gamification mechanisms described by Werbach and Hunter such as challenges, cooperation and competition, feedback, rewards, and victory states [27].

### Measures

Knowledge of pain was measured using the “Neurophysiology of Pain Questionnaire” developed by Professor Moseley [32] in its version adapted to Spanish [33]. The questionnaire consists of 19 questions requiring true, false, or undecided answers, with 19 points as the maximum score. The questionnaire proved to be easy to use, short, valid, and reliable [33].

Another measurement instrument used to assess misconceptions about pain and movement was the “Tampa Scale for Kinesiophobia” (TSK) developed by R. Miller and S. Kopri in the early 1990s, as revised and shortened from the TSK-11 questionnaire by Woby et al. [34]. This questionnaire was validated in Spanish by Gómez et al. [35]. It has 11 questions to be answered on a scale from 1 (totally disagree) to 4 (totally agree), with scores ranging from 11 to 44 points. The questionnaire has proven reliable, valid, and easy to use [36].

The students’ motivation and satisfaction with the methodology used were measured using an adaptation of Escobar-Pérez and Lobo-Gallardo [37]. This questionnaire consists of 16 questions to be answered using the Likert scale. It comprised two questions with five levels of response that assessed the degree of usefulness of the methodology and the subject’s content; 14 questions with six levels of response that assessed the degree of student compliance with the statements made; and a single question answered with 11 levels of response that assessed the likelihood of recommending the web platform to future students.

### Statistical analysis

The variables of gender, age, grade studied, and whether they had ever suffered pain were analysed. The results of the sociodemographic variables are presented in Table 2.

**Table 2:** Sociodemographic variables

*Variables*	Modalities	Percentage (%)
***Gender***	Male	50
	Female	50
***Age***	20	35,5
	21	30
	22	8,3
	23	5
	24	5
	25	6,7
	26	3,3
	28	1,7
	31	1,7
	33	1,7
	40	1,7
***Degree***	Physiotherapy	31,7
	Physiotherapy-Sports Sciences	31,7
	Physiotherapy-Nursing	36,7
***Pain***	Yes	80
	No	20

The main variable (knowledge of chronic pain) and the secondary variable (erroneous knowledge of pain and movement) are expressed with the mean (standard deviation) and the effect size (d). The effect size was determined using the “barometer of influences” [38]. Thus, an effect size below 0 is associated with an inverse effect; 0 to 0.15 with a developmental effect; 0.16 to 0.40 with a typical teacher effect; and 0.40 to 1.20 is associated with the desired effect area.

To analyse the outcomes of pain neurophysiology knowledge and kinesiophobia, the authors used Student’s t-test for related samples. Percentages were used to analyse the students´ satisfaction and motivation with the gamified web platform.

The statistical power was calculated with the GRANMO sample calculator. The statistical analysis was carried out with SPSS software, assuming an alpha error of 0.05.

### Ethics approval and consent to participate

All participants provided their written consent after being informed about the objectives of the study and guaranteeing their confidentiality of personal data according to Regulation (EU) 2016/679 of the European Parliament and Council of 27 April 2016.

This study followed the Declaration of Helsinki principles and Good Clinical Practice Guidance (CPMP/ICH/135/95), and was approved by the Studies and Research Committee of the Faculty of Nursing and Physiotherapy of the University of Lleida.

## Results

Regarding the sample subjects, it should be noted that the proportion of male and female students is identical. Most of the students were in the age range of 20 to 21 years, with a mean age of 22,22 (SD3,5). Additionally, 80% of the students stated that they had suffered pain at some time, and 20% had never suffered pain (Table 2).

Pain neurophysiology knowledge was assessed with the “Neurophysiology of Pain Questionnaire”. The results show that there is a statistically significant influence regarding the use of the gamified web platform on improvement in knowledge of chronic pain [t(59) = 5.87, *p* < 0.01], with an effect size of d = 0.76, placing it within the desired effect zone established by Hattie [39] in the educational field (Table 3).

**Table 3:** Results on knowledge about chronic pain

Neurophysiology of Pain Questionnaire		
	**Mean (standard deviation)**	**Effect size (d)**
***Pre- intervention***	8,8 (2,22)	
***Post-intervention***	10,18 (1,87)*	0,76
**TSK-11**		
	**Mean (standard deviation)**	**Effect size (d)**
***Pre- intervention***	32,98 (4,76)	
***Post-intervention***	28,35 (7,21)*	0,6

^*^p < *0,01*

The secondary variable on erroneous beliefs was assessed with the “TSK-11” questionnaire. The results show that there is a statistically significant influence of the use of the gamified web platform on the fear of being injured by movement [t(59) = -4.67, *p* < 0.01], with the effect size being d = 0.6, which is within the zone of the desired effect established by Hattie (2015) (Table 3).

Once the study was completed, the statistical power was calculated. For the questionnaire on the neurophysiology of pain, accepting an alpha risk of 0.05 in a two-tailed test with 60 subjects, the statistical power was 94% for detecting as statistically significant the difference between the means of 8.8 for the pre-intervention measurement and 10.18 for the post-intervention measurement.

The statistical power for the TSK-11 questionnaire, accepting an alpha risk of 0.05 in a bilateral contrast with 60 subjects, was 100% for detecting as statistically significant the difference between the mean of 32.98 of the pre-intervention measurement and 28.35 for the post-intervention measurement.

The questionnaire results in terms of evaluating student motivation and satisfaction with the methodology showed that 95% of the students considered the subject of physiotherapy in clinical specialties as useful for their future profession.

Seventy percent of the students stated that the activities carried out on the gamified web platform helped them understand the concepts explained. Therefore, 71.7% of the students believed that the experience was worth the time spent on the subject.

A total of 58.4% of the students thought that the methodology used made the classes more interesting. The same percentage of students believed that the methodology helped them learn more during the teaching–learning sessions and that it had helped them modify their view of the student’s role as a passive recipient of information.

A total of 56.6% of the students felt more involved in the subject than if another teaching–learning methodology had been used, and 51.7% expressed that it encouraged them to ask questions and engage in discussions.

A total of 73.3% of the students considered that this methodology showed interest on the part of the teacher in teaching. In addition, 53.3% of the students considered that generalizing this activity to other subjects would improve university teaching quality.

On the other hand, 70% of the students considered the subject's content improved thanks to its methodology.

A total of 63.3% of the students believed that the methodology facilitated using ideas and information they already knew to understand something new. On the other hand, 66.6% of the students considered that the methodology helped them relate what they learned in this subject with other subjects.

Concerning motivation, 66.6% of the students thought that the tools used in this subject motivated them and helped them review content. Sixty-five percent of students thought that the methodology increased their motivation in terms of the subject's content, and 60% felt more motivated to participate. Therefore, 71.7% of the students would probably recommend that physical therapy students who want to learn pain neurophysiology enrol in the teaching–learning sessions using this methodology.

## Discussion

Our results suggest that using a gamified web platform effectively increases knowledge of the neurophysiology of pain and reduces misconceptions about pain and movement among students in UdL’s physiotherapy department. Concerning the satisfaction and motivation with the methodology used, the results showed that it has been useful for increasing students' motivation and satisfaction. Therefore, using these tools effectively promotes students’ learning in a subject as complex as the neurophysiology of chronic pain. It is possible to increase student knowledge and improve their motivation and satisfaction in the teaching–learning process.

Our findings coincide with those found by Villalustre and del Moral [25] in their study on the use of gamification, in which the students had to carry out a socio-educational project with collaborative learning. The study, conducted with 161 students of the Degree of Pedagogy (University of Oviedo), showed that more than 70% of the students obtained a high level of satisfaction with the methodology used and that this methodology managed to facilitate learning and increase student motivation. In line with these results, the study by Corchuelo-Rodriguez [26], in which the subject “Basic Digital Competence” at the University of La Sabana, Colombia, was gamified with a sample of 89 undergraduate students, of which 35 were health sciences students from the disciplines of physical therapy, medicine, psychology, and nursing, was able to motivate students by 88% and increase their participation in the subject. Likewise, Domínguez et al. [30], in their study on gamified learning experiences, concluded that gamified web platforms used in a learning environment are useful for increasing student motivation. However, they found no significant differences concerning learning between the group that followed the traditional learning methodology and those who used the gamified e-learning platform. This study was carried out with university students from different disciplines in the course “Qualification for ICT Users”. It is interesting to note that competing publicly with their peers was not at all interesting or motivating and became discouraging for several subjects of the study. On the other hand, Watt-Watson et al. [22] conducted a study on students from their university to increase knowledge about pain and reduce erroneous beliefs. To do this, they mainly used the simulation of a clinical case through videos, from which they obtained a significant increase in knowledge about pain. All of this demonstrates that the methodology employed by Watt-Watson et al. [22] and the present study has proven to be effective, offering encouraging results.

Although, as mentioned above, about 65% of our students showed high levels of motivation, for 35% of the students, this has not been a motivating experience. In the study of Domínguez et al. [30], 56 students found that traditional activities were more motivating than gamified activities. Similarly, we could hypothesize that some of the students of our study may find traditional teaching activities more motivating than gamified activities. These results suggest that, in future investigations, it would be interesting to optimize the motivation and satisfaction questionnaire to determine with greater precision which activities and sections of the entire methodology are employed to increase the students' motivation and which do not or in what proportion each one is increased.

As has been noted, and coinciding with the observations of Villalustre Mart and del Moral Pérez [25] and of Domínguez et al. [30], the creation of a recreational experience for students is the result of important cognitive activity on the part of teachers and their collaborators, since it requires high doses of imagination and creativity. At the same time, a great effort had to be made to make the experience motivating and maintain the main objective of the training in specific content, in this case, the neurophysiology of pain.

It should be noted that an increase in knowledge of the neurophysiology of pain among future health professionals would lead to a better approach to patients' chronic pain. This could improve patients' quality of life, decreasing their need for accessing the health system, and consequently reducing these patients' health costs.

Compared to a more traditional education, a gamified website has advantages and weaknesses. As shown in our results, for a high percentage of our students and students of other studies [25, 26], gamification helps to increase knowledge and motivation and contributes to learning through game enjoyment. However, compared to traditional face-to-face learning, gamification needs interaction and collaboration with specialists in information technology, so teaching staff could see gamification as time-consuming, not to mention the cost of the audiovisual material and the implementation of gamification mechanics. Considering the latter, it would be interesting to include cost-effectiveness measures in future studies.

A limitation of the present study is the impossibility of comparing the results with a control group. Thus, possible differences between the face-to-face model and the one used in this project cannot be determined. On the other hand, it would be interesting to evaluate whether the knowledge acquired by these students is maintained over time and whether they manage to improve their approach to chronic pain in their patients. Likewise, future similar studies could be carried out in students of other health sciences disciplines to improve their approaches for patients suffering from chronic pain. The possible practical implications of our study have been summarised in Table 4.

**Table 4:** Practical implications

Practice points
• The limited amount of pain-related training in most healthcare grades curricula leads to a significant lack of knowledge
• The lack of knowledge about the neurophysiology of pain may lead to difficulties in managing patients with chronic pain
• Our gamified web platform has proven to be a valid teaching–learning method for pain neurophysiology
• Using a gamified web platform effectively increases the knowledge about the neurophysiology of pain in Physiotherapy students
• This teaching strategy has also been shown to be effective in increasing our students' motivation and satisfaction

## Conclusions

In conclusion, using a gamified web platform effectively increases student knowledge of the neurophysiology of pain. This gamified web platform has also been shown to be effective in improving students' motivation and satisfaction in the Degree in Physiotherapy of the UdL on learning about the neurophysiology of pain. Likewise, more research is needed on this methodology to create more powerful and motivating tools that help in the transfer of knowledge to our students and thus obtain better-prepared health professionals who demonstrate a better approach for their patients with chronic pain. 

## Data Availability

The datasets used and/or analysed during the current study are available from the corresponding author on reasonable request.

## Abbreviations

CLBP: Chronic Low Back Pain
FMS: Fibromyalgia Syndrome
IASP: Association for the Study of Pain
ICTs: Information and Communication Technologies
TSK: Tampa Scale for Kinesiophobia
UdL: University of Lleida
